# Identification and validation of a diagnostic and prognostic model based on immune escape and cancer-associated fibroblast-related genes in lung adenocarcinoma

**DOI:** 10.1097/MD.0000000000045756

**Published:** 2025-11-14

**Authors:** Yuhui Ma, Xu Li, XueNa Wang, Bin Song, Rui Geng, Yuan Hao, Wen Su

**Affiliations:** a Department of Cancer Center, Third Hospital of Shanxi Medical University, Taiyuan, Shanxi Province, China; b Department of Thoracic Surgery, Shanxi Bethune Hospital, Shanxi Academy of Medical Sciences, Taiyuan, Shanxi Province, China; c Department of Cancer Center, Shanxi Bethune Hospital, Shanxi Academy of Medical Sciences, Taiyuan, Shanxi Province, China; d Department of Research-Oriented Ward, Yuncheng Central Hospital Affiliated to Shanxi Medical University, Yuncheng, Shanxi Province, China; e Department of Clinical Trials Center, Shanxi Province Cancer Hospital, Shanxi Hospital Affiliated to Cancer Hospital, Chinese Academy of Medical Sciences, Cancer Hospital Affiliated to Shanxi Medical University, Taiyuan, Shanxi Province, China; f Department of Medical Laboratory, Cancer Hospital Affiliated to Shanxi Medical University, Shanxi Province Cancer Hospital, Taiyuan, Shanxi Province, China.

**Keywords:** cancer-associated fibroblasts, drug sensitivity, immune escape-related genes, lung adenocarcinoma, risk model

## Abstract

Lung adenocarcinoma (LUAD), a common type of non-small cell lung cancer, is associated with low survival rates and challenges in early detection. Therefore, identifying prognostic biomarkers is crucial for improving patient outcomes. This study utilized 2 datasets – the Cancer Genome Atlas-Lung Adenocarcinoma (TCGA-LUAD) and GSE72094 – along with 182 immune escape-related genes and 597 cancer-associated fibroblast-related genes. Weighted gene co-expression network analysis was used to identify module genes. Differential expression analysis of TCGA-LUAD data revealed LUAD-associated differentially expressed genes, which were then intersected with module genes to identify LUAD-specific differentially expressed immune escape-cancer fibroblast-related genes. To identify potential biomarkers and develop a risk model, univariate Cox regression, least absolute shrinkage and selection operator analysis, and multivariate Cox regression were performed. Gene Ontology and Kyoto Encyclopedia of Genes and Genomes databases were used for enrichment analysis. Immune infiltration and immune cell-biomarker correlations were assessed using CIBERSORT, and the pRRophetic tool was employed to predict LUAD chemotherapeutic sensitivities. Reverse transcription-quantitative polymerase chain reaction was used to validate the expression of prognostic genes in non-small cell lung cancer. The results showed that 183 differentially expressed immune escape-cancer fibroblast-related genes were identified by intersecting 1460 module genes with 5439 differentially expressed genes. Six genes (*KRT8*, *S100A16*, *COL4A3*, *SMAD9*, *MAP3K8*, and *CCDC146*) were selected as potential biomarkers for risk modeling. Gene Ontology enrichment analysis highlighted the involvement of glucose metabolism, ion channel complexes, and channel activity-related genes. Kyoto Encyclopedia of Genes and Genomes analysis revealed pathways related to morphine addiction and protein digestion/absorption. Immune infiltration analysis identified significant differences in 9 immune cell types, including memory B cells and CD8 T cells, between risk groups. Sensitivity to chemotherapeutics, such as AZD6482, ABT-263, A-770041, and BMS-536924, was observed in LUAD. Reverse transcription-quantitative polymerase chain reaction validation results demonstrated that *KRT8* and *S100A16* were significantly upregulated in tumor tissues, while *COL4A3* and *SMAD9* expression was downregulated, which was consistent with the TCGA-LUAD database analysis. In conclusion, 6 genes (*KRT8*, *S100A16*, *COL4A3*, *SMAD9*, *MAP3K8*, and *CCDC146*) were identified as potential biomarkers, offering valuable insights into LUAD pathogenesis and therapeutic strategies.

## 1. Introduction

Lung cancer remains the leading cause of cancer-related mortality worldwide, with an estimated 1.8 million deaths annually.^[1]^ Non-small cell lung cancer (NSCLC) represents approximately 85% of lung cancer cases, while SCLC accounts for around 15%.^[2]^ Lung adenocarcinoma (LUAD), the most common histological subtype, constitutes approximately 40% of all lung cancer cases and 60% of NSCLC cases.^[3]^ The etiology of LUAD is complex and multifactorial, involving genetic mutations such as EGFR and KRAS, smoking, and environmental factors like air pollution.^[4]^ Despite advances in targeted therapies and immunotherapies, the 5-year survival rate for LUAD remains low, with most patients diagnosed at advanced stages.^[5]^ Although PD-1/PD-L1 inhibitors have improved prognosis in some patients, the response rate remains under 20%, and the predictive value of existing biomarkers like PD-L1 expression and tumor mutational burden (TMB) is limited.^[6]^ Immune escape mechanisms play a significant role in limiting therapeutic effectiveness, contributing to tumor recurrence and poor prognosis.^[7]^ Consequently, identifying novel prognostic biomarkers, particularly those linked to immune escape in LUAD, is essential for enhancing diagnostic accuracy and treatment outcomes.

Tumor cells employ various strategies to evade immune system recognition and attack, which allows their survival and proliferation, thus promoting rapid tumor progression and deterioration.^[8]^ Recent studies on immune escape mechanisms in LUAD have focused on the regulation of immune checkpoint molecules and the TME.^[9]^ B4GALT1 enhances immune escape by stabilizing PD-L1 through regulation of its N-glycosylation.^[10]^ Zhu et al utilized spatial transcriptomics and single-cell RNA sequencing to examine the dynamic progression of LUAD from adenocarcinoma in situ to invasive adenocarcinoma.^[11]^ Their research highlighted the pivotal role of the TGF-β signaling pathway and the UBE2C+ cancer cell subpopulation in LUAD invasion.^[11]^ These findings highlight the complexity of immune escape, involving interactions between immune cells and tumor cells, as well as TME remodeling, offering potential targets and novel directions for personalized LUAD diagnosis and therapy.

Ongoing research into tumor immune evasion mechanisms has revealed that dynamic alterations in cellular components (CCs) and cytokines within the TME are crucial for tumor immunoediting.^[12]^ As key elements of the TME, cancer-associated fibroblasts (CAFs) play multifaceted roles in tumor progression, immune evasion, and metabolic reprogramming.^[13]^ One study demonstrated that the CAF-specific lncRNA LINC01614 promotes glutamine uptake by LUAD cells through exosomal delivery, thereby facilitating tumor growth and metastasis. This finding highlights the therapeutic potential of targeting CAF-specific lncRNAs to block glutamine influx and attenuate LUAD tumor activity.^[14]^ Furthermore, gene editing strategies that suppress HNRNPK expression in CAFs and reduce CLCN3 secretion can inhibit TGF-β1 production, further curbing LUAD progression.^[15]^ Despite the established role of CAFs in LUAD, their heterogeneity and plasticity present significant challenges for targeted drug delivery.^[16]^ Thus, understanding the interplay between CAFs and immune escape mechanisms is vital for optimizing LUAD treatment strategies.

This study aimed to identify LUAD prognostic genes associated with immune evasion and CAFs by integrating data from public databases and applying various bioinformatics analysis methods. The resulting genes were used to construct a risk prediction model. In resource-limited regions (such as primary healthcare settings or developing areas), the application of high-throughput sequencing and multi-omics analysis is often hindered by factors such as cost and technical barriers, limiting the use of traditional diagnostic methods and prognosis assessments.^[17]^ Our research presents a cost-effective and efficient alternative for studying the immune escape mechanisms in LUAD. The development of this model can provide early prognostic predictions, enabling the formulation of more precise and personalized treatment strategies, advancing the field of immunotherapy, and ultimately improving the prognosis of patients with LUAD.

## 2. Materials and methods

### 2.1. Ethics declarations

This study received approval from the Medical Ethics Committee of Shanxi Bethune Hospital (Shanxi Academy of Medical Sciences; No. LYLL-2024-003/PJ01) and was conducted in accordance with the principles outlined in the Declaration of Helsinki. All participants provided written informed consent. The research was carried out at the Department of Cancer Center, Shanxi Bethune Hospital, Xiaodian District, Taiyuan, China.

### 2.2. Data source

The cancer genome atlas-lung adenocarcinoma (TCGA-LUAD) dataset, containing complete survival data, was accessed via the University of California Santa Cruz Xena database (https://xenabrowser.net/) and includes 526 LUAD and 59 normal samples. The GSE72094 dataset was retrieved from the gene expression omnibus database (https://www.ncbi.nlm.nih.gov/geo/) and consists of 442 LUAD samples. Based on previous research,^[18]^ 182 immune escape-related genes (IERGs) were obtained. Additionally, the Molecular Signatures Database (https://www.gsea-msigdb.org/) provided 597 cancer-associated fibroblast-related genes (CAFRGs).

### 2.3. Calculation of single-sample gene set enrichment analysis (ssGSEA) score

Initially, univariate Cox regression analysis was conducted on the TCGA-LUAD gene expression matrix using the survival package (v3.3-1) to identify LUAD prognosis-associated genes. Next, single-sample gene set enrichment analysis was applied to calculate the CAF score and IERG-score for each sample, using the 182 IERGs and 597 CAFRGs.^[19]^ For survival analysis, the optimal cutoff value was automatically determined using the cutpointr function from the Survminer package. Based on this, patients were categorized into low- and high-score groups. Kaplan–Meier (K–M) curves were used to visualize survival differences, with statistical significance assessed by the log-rank test.^[20]^

### 2.4. Identification of key gene modules by weighted gene co-expression network analysis (WGCNA)

To identify key modules in TCGA-LUAD, sample clustering was performed using weighted gene co-expression network analysis (WGCNA).^[21]^ The samples underwent hierarchical clustering, and outliers were removed. The optimal soft threshold (β) was selected based on the first instance where the scale-free topology fit index reached 0.85. A β value of 6 was chosen to construct the gene co-expression network. Gene modules were defined using the hybrid dynamic tree cutting algorithm, and IERG-score and CAF-score were treated as phenotypic traits. Pearson correlation analysis was performed to evaluate the correlation between phenotypic traits and gene modules. The key module was defined as the 1 with the highest correlation to either the IERG-score or CAF-score, and the genes within this module were designated as key module genes.

### 2.5. Identification and enrichment analysis of key genes

In the TCGA-LUAD dataset, differentially expressed genes (DEGs) were identified using DESeq2 (v 1.34.0), with filtering criteria of |log_2_ FC| > 1 and *P*-value <.05.^[22]^ A set of genes associated with immune escape and CAFs in LUAD (DE-IE-CAF-RGs) was obtained by intersecting these DEGs with the modular genes. Kyoto Encyclopedia of Genes and Genomes (KEGG) and gene ontology (GO) enrichment analyses were conducted on the DE-IE-CAF-RGs using the clusterProfiler package (v. 4.6.0).^[23]^ A protein–protein interaction network was constructed using the STRING database (https://string-db.org/) with a confidence threshold of 0.15.^[24]^

### 2.6. Construction and validation of risk models

Using the TCGA-LUAD dataset, this study identified prognostic genes and constructed a risk model through multivariate Cox regression, least absolute shrinkage and selection operator (LASSO) regression, and univariate Cox regression. Initially, univariate Cox regression was applied to select significant genes, followed by LASSO regression to minimize the number of genes and prevent overfitting. Ultimately, a multivariate Cox regression model was constructed, and the risk score for each patient was calculated using the following formula: $Risk\;score=\sum {\mathrm{\backslash nolimits}}_{i=1}^{n}\left(coef\;i\;*\;Xi\right)$.^[25]^ Patients were then divided into high- and low-risk groups based on the median risk score. K–M survival curves were used to compare survival between the 2 groups. The model’s predictive performance was validated by calculating the area under the receiver operating characteristic (ROC) curve using the survival ROC package (v1.0.3).^[26]^ To further assess the robustness of the model, the validation set (GSE72094) was analyzed using the same methodology.

### 2.7. Prognostic nomogram development and validation for clinical outcome prediction in LUAD

Expression heatmaps of prognostic genes across various clinical features were generated using the ComplexHeatmap R package.^[27]^ Variations in risk scores among different clinical features were evaluated using the Wilcoxon test. Multivariate and univariate Cox analyses, incorporating both risk scores and clinical features, were performed to identify independent prognostic factors. All factors were then tested for the proportional hazards assumption, and a prognostic model was constructed.^[28]^ Additionally, a nomogram was developed using the RMS R package (v 6.5.0).^[29]^ The nomogram was further validated through K–M survival analysis, and its accuracy was assessed by calibration and ROC curves.

### 2.8. Tumor microenvironment analysis

The CIBERSORT algorithm was applied to evaluate the abundance of 22 immune cell types infiltrating TCGA-LUAD.^[30]^ Differences in immune cell abundance between groups were assessed using the Wilcoxon test.^[31]^ Spearman correlation analysis was employed to explore the relationship between prognostic genes and differential immune cells.^[32]^ Additionally, the differences in the 21 major histocompatibility complex (MHC) genes between risk groups were tested using the Wilcoxon test, based on previous studies.^[33]^ Furthermore, the tumor immune dysfunction and exclusion (TIDE) score for each patient in TCGA-LUAD was calculated, and Spearman correlation analysis was performed between TIDE and risk scores. Wilcoxon tests were used to evaluate score differences between the low- and high-risk groups.

### 2.9. Analysis of tumor mutational burden (TMB)

TMB scores were calculated for each patient in TCGA-LUAD, and differences were explored using TMB scores.^[34]^ Survival analysis of patients with LUAD was performed by stratifying them into low- and high-TMB groups based on the median TMB score, followed by K–M survival analysis. Survival outcomes were further analyzed by combining risk groups (low and high) with TMB groups (low and high), generating 4 subgroups: HighRisk-HTMB, HighRisk-LTMB, LowRisk-HTMB, and LowRisk-LTMB.

### 2.10. Drug sensitivity

The pRRophetic tool was employed to calculate the half-maximal inhibitory concentration of each drug for every sample in the TCGA-LUAD dataset. Half-maximal inhibitory concentration differences were compared using Wilcoxon tests.^[35]^

### 2.11. Analysis of gene mutations

Mutation data from TCGA were utilized for mutational analysis using Maftools (v 2.10.5).^[36]^ The top 20 genes with the highest mutation frequencies were selected to examine their mutation status.

### 2.12. Gene set enrichment analysis

Gene set enrichment analysis (GSEA) was performed on DEGs between the low- and high-risk groups using the “GOc5.all.v7.1.symbols.gmt” and “c2.cp.kegg.v7.1.symbols.gmt” gene sets, which were sourced from the molecular signatures database.^[37]^

### 2.13. Quantitative real time polymerase chain reaction (qRT-PCR)

Quantitative real time polymerase chain reaction (qRT-PCR) was performed to analyze RNA expression in LUAD tumors and their adjacent normal tissues to validate the expression of prognostic genes. Tissue samples were collected from 6 newly diagnosed patients with LUAD who underwent surgical resection at the Department of Thoracic Surgery, Shanxi Bethune Hospital. All patients had LUAD confirmed through pathological diagnosis, with no history of smoking or preoperative radiotherapy or chemotherapy. Frozen samples were processed with TRIzol reagent (Ambion, China) for total RNA extraction. Reverse transcription was conducted to convert equal amounts of mRNA into complementary DNA (cDNA). The 2× Universal Blue SYBR Green qPCR Master Mix kit (Servicebio, China) was used for qPCR. The 2^−ΔΔCT^ method was applied to quantify gene expression, using GAPDH as the reference control. Primer sequences for the genes are provided in Table S1, Supplemental Digital Content, https://links.lww.com/MD/Q616. This study was conducted in accordance with the principles of the Declaration of Helsinki. Approval was granted by the Medical Ethics Committee of the Shanxi Bethune Hospital (Shanxi Academy of Medical Sciences; No. LYLL-2024-003/PJ01). Approval date: June 14, 2024.

### 2.14. Statistical analysis

Bioinformatics analysis was conducted using R software (v. 4.2.2). Statistical significance was determined at *P* < .05.

## 3. Results

### 3.1. Identification of key module genes

**Figure 1.**
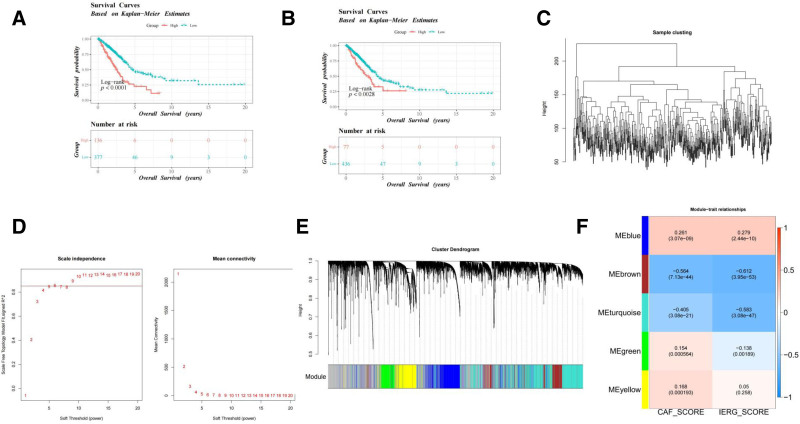
Identification of key module genes by WGCN. (A, B) K–M survival curves for the low and high IERG score groups (A), as well as the high- and low-CAF score groups (B). (C) Sample clustering analysis to detect outliers. (D) Soft-threshold power selection for WGCNA. (E) Module identification associated with IERG-score and CAF-score. (F) Correlation heatmap between gene modules and IERG-score/CAF-score. CAF = cancer-associated fibroblast, IERG = immune escape-related gene, K–M = Kaplan–Meier, WGCNA = weighted genetic co-expression network analysis.

To investigate the impact of immune escape and CAFs on LUAD prognosis, the IERG-score and CAF-score were calculated, followed by survival and co-expression network analyses to identify key gene modules. K–M survival analysis stratified by the IERG-score and CAF-score is presented in Figure 1A, B, respectively. Patients with a high IERG score exhibited significantly lower overall survival compared to those with a low score (log-rank *P* < .0001). Similarly, a high CAF score was associated with poorer overall survival (log-rank *P* = .0028). To further explore genes linked to the IERG-score and CAF-score groups, WGCNA was performed. No outliers were detected in the sample clustering results (Fig. 1C). A soft threshold of 6 was selected, where *R*^2^ approached the 0.85 threshold (red line), ensuring a scale-free distribution as closely as possible (Fig. 1D). Six gene modules were ultimately identified (Fig. 1E). Pearson’s correlation analysis revealed that the brown module (IERG-score: cor = −0.612, CAF-score: cor = −0.564) had the strongest correlation with the 2 traits (*P* < .05), containing 1460 key module genes for subsequent analyses (Fig. 1F). In conclusion, WGCNA identified gene modules strongly associated with immune escape and CAF activity, providing a basis for constructing a prognostic model.

### 3.2. *Screening and functional enrichment analysis of DE*-IE-*CAF-RGs*

**Figure 2.**
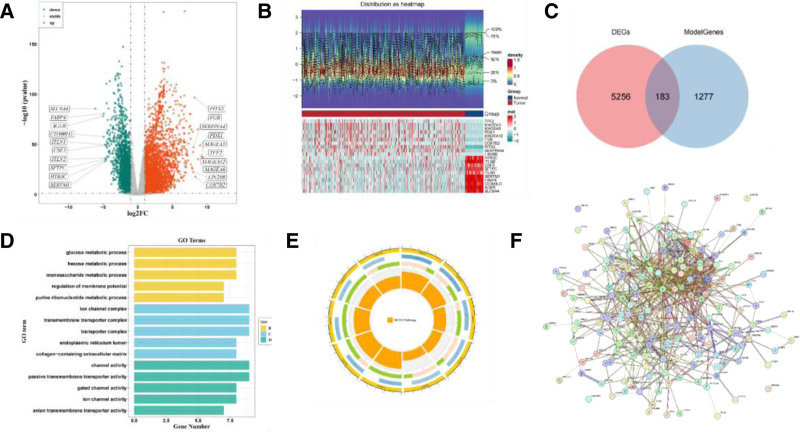
Identification and functional enrichment analysis of DE-IE-CAF-RGs. (A) Volcano plot of differential genes. The abscissa represents the log FC value, and the ordinate represents −log_10_ (*P* value). Red dots represent upregulated genes, green dots represent downregulated genes, and gray dots represent genes with no significant difference in expression. (B) Heatmap of differential genes. The color represents the relative expression level of genes: red indicates a relatively high expression level, and blue indicates a relatively low expression level. (C) Venn diagram showing the overlap between the WGCNA modular genes and DEGs. (D) GO enrichment analysis for DE-IE-CAF-RGs. (E) KEGG enrichment analysis of DE-IE-CAF-RG. (F) PPI networks of DE-IE-CAF-RG. Nodes represent genes, lines represent interaction relationships, and the color of lines represents prediction methods. DEGs = differentially expressed genes, DE-IE-CAF-RGs = differentially expressed immune escape-cancer fibroblast-related genes, GO = gene ontology, KEGG = Kyoto Encyclopedia of Genes and Genomes, PPI = protein–protein interaction, WGCNA = weighted genetic co-expression network analysis.

To further investigate the genes and functions linked to immune escape and CAF activity, key genes were identified through differential expression and co-expression network analyses, followed by functional enrichment analysis. Initially, 5439 DEGs associated with LUAD were identified, of which 3472 were upregulated and 1947 were downregulated (Fig. 2A, B). These DEGs were intersected with genes from the brown module identified in the WGCNA, resulting in 183 DE-IE-CAF-RGs (Fig. 2C). GO analysis revealed that these genes were primarily involved in biological processes (BPs) such as glucose metabolism and hexose metabolism, CCs like ion channel complexes, and molecular functions including passive transmembrane transport and channel activity (Fig. 2D). KEGG pathway analysis identified 80 significantly enriched signaling pathways, including morphine addiction, and protein digestion and absorption (Fig. 2E). Additionally, a protein–protein interaction network comprising 621 edges and 166 nodes was constructed, highlighting key interactions such as GABRE-GABRR2 and WDR88-ANKRD11 (Fig. 2F). Overall, the 183 DE-IE-CAF-RGs contribute to several key signaling pathways and metabolic processes, offering valuable insights into the immune escape mechanisms of LUAD.

### 3.3. Construction of prognostic risk model based on prognostic genes and its validation in LUAD

**Figure 3.**
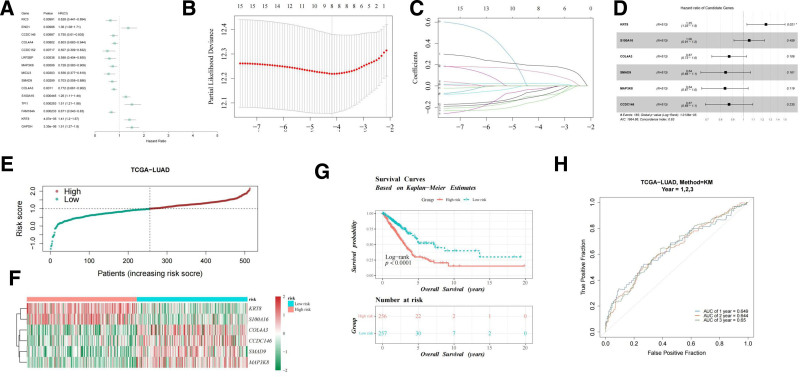
Construction of the risk model in the TCGA-LUAD dataset. (A) Identification of 15 genes through univariate Cox analysis. (B, C) Identification of 6 key genes as independent prognostic factors by LASSO and (D) multivariate Cox regression analysis. (E) Risk score of the training set. The abscissa represents patient samples sorted by risk score, with risk scores increasing from left to right; the ordinate represents risk scores; the horizontal dashed line represents the optimal cutoff of risk scores; and the vertical dashed line represents the sample corresponding to the median risk score. (F) Heatmap representation of 6-gene expression patterns in low- and high-risk groups. Colors represent the relative expression levels of genes, with red indicating relatively high expression levels and blue indicating relatively low expression levels. (G) K–M survival curves. The K–M curve consists of 2 parts. The upper part is a graph of survival time and survival probability, where the abscissa represents the total survival time of the samples and the ordinate represents the survival probability. The red curve indicates the high-risk group, and the blue curve indicates the low-risk group. The lower part is a risk table, showing the number of remaining samples in different groups at different survival times. (H) ROC curves of risk model predicting 5-yr, 3-yr, and 1-yr overall survival. K–M = Kaplan–Meier, LASSO = least absolute shrinkage and selection operator, ROC = receiver operating characteristic, TCGA-LUAD = the cancer genome atlas-lung adenocarcinoma.

To identify key genes associated with LUAD prognosis, univariate Cox regression analysis was performed, resulting in the selection of 15 significant genes. These genes were then used to establish a risk prediction model (Fig. 3A). LASSO regression analysis narrowed down the selection to 8 key genes (Fig. 3B, C). Ultimately, 6 prognostic genes – KRT8, S100A16, COL4A3, SMAD9, MAP3K8, and CCDC146 – were identified through multivariate Cox regression analysis and utilized to construct the risk model (Fig. 3D). Patients were classified into low-risk (n = 257) and high-risk (n = 256) groups based on the median risk score. A risk curve was plotted (Fig. 3E), and a heatmap of the 6 genes is presented in Figure 3F. K–M survival analysis showed that the high-risk group had significantly lower survival rates compared to the low-risk group (log-rank test *P* < .0001; Fig. 3G). The performance of the model was evaluated using area under curve (AUC) values from the TCGA-LUAD training set, which consistently exceeded 0.6, indicating strong predictive capability at various time points (Fig. 3H).

**Figure 4.**
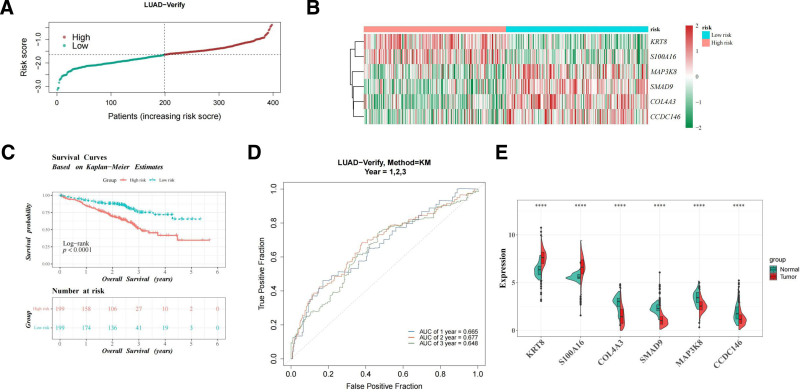
Validation of risk model in GSE72094 dataset. (A) Risk score distribution in high- and low-risk groups. The abscissa represents patient samples sorted by risk scores, with risk scores increasing from left to right; the ordinate represents risk scores; and the dashed line indicates the median risk score and the corresponding number of patients. (B) Heatmap showing the expression patterns of the 6 genes in both groups. Colors represent the relative expression levels of genes: red indicates a relatively high expression level, and blue indicates a relatively low expression level. (C) The K–M survival curve showing worse survival in the high-risk group. The K–M curve consists of 2 parts. The upper part is a graph depicting survival time and survival probability, where the abscissa represents the total survival time of the samples, and the ordinate represents the survival probability. The red curve indicates the high-risk group, while the blue curve indicates the low-risk group. The lower part is a risk table, showing the number of remaining samples in different groups at various survival times. (D) ROC curves for predicting 5-yr, 3-yr, and 1-yr survival and (E) six gene expression levels. ****P < .0001. K–M = Kaplan–Meier, ROC = receiver operating characteristic.

The risk model was subsequently validated using the GSE72094 dataset. The risk curve and gene heatmap for the GSE72094 dataset are shown in Figure 4A, B. As with the TCGA-LUAD data, the high-risk group in GSE72094 demonstrated significantly lower survival rates than the low-risk group, as confirmed by the K–M survival curve (log-rank test *P* < .0001; Fig. 4C). ROC curve analysis yielded AUC values exceeding 0.6 for 5-year, 3-year, and 1-year survival (Fig. 4D). Moreover, gene expression analysis showed that the expressions of KRT8 and S100A16 were significantly increased in tumor tissues, while the expressions of COL4A3, SMAD9, MAP3K8 and CCDC146 were decreased (Fig. 4E). These differential expression patterns suggest that the identified genes may play pivotal roles in LUAD initiation and progression.

### 3.4. Nomogram construction based on risk model and clinical features

**Figure 5.**
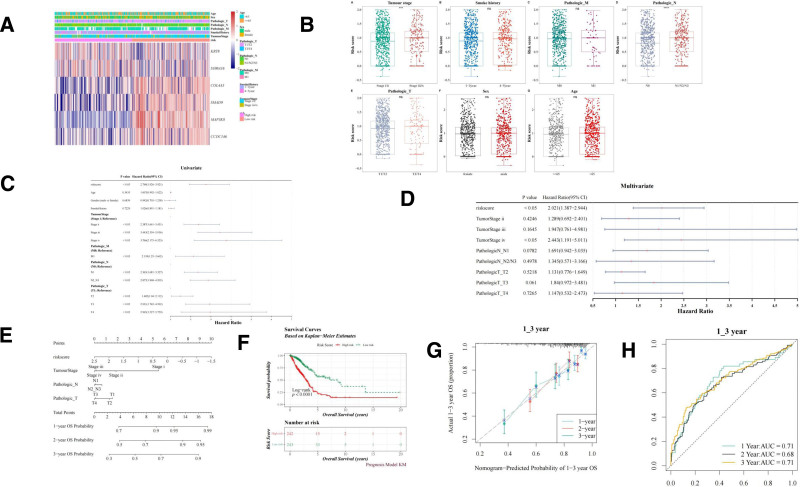
Independent prognostic analysis for LUAD. (A) Heatmap illustrating prognostic gene expression across different clinical features. (B) Clinical feature distribution in low- and high-risk groups. ns; ***P < .001; and *****P < .00001. (C, D) Assessment of clinical features and risk score via univariate (C) and multivariate Cox regression (D). (E) Nomogram predicting 5-, 3-, and 1-yr survival. (F) K–M survival curve for nomogram-based risk groups. (G) Nomogram calibration curve. (H) ROC curve for nomogram. K–M = Kaplan–Meier, LUAD = lung adenocarcinoma, ns = not significant, ROC = receiver operating characteristic.

Further survival analysis and nomogram construction incorporating clinical features and risk scores were performed to validate the clinical relevance of the risk model. Heatmaps depicting the expression of prognostic genes in low- and high-risk groups across different clinical features are shown in Figure 5A. Figure 5B illustrates significant variations in risk scores across different tumor stages, M stage, and N stage. Univariate Cox regression analysis revealed a strong association between survival and pathological stages (M, N, and T stages), tumor stage, and risk score (Fig. 5C). Multivariate Cox regression analysis identified pathological stages N and T, along with the risk score, as independent prognostic factors influencing survival (Fig. 5D). A nomogram incorporating these factors was developed to predict 5-, 3-, and 1-year survival rates (Fig. 5E). The K–M survival curves further demonstrated that the high-risk group had significantly poorer survival than the low-risk group (Fig. 5F). The nomogram’s predictive accuracy for 5-, 3-, and 1-year survival was confirmed by the calibration curve (Fig. 5G), and the AUC values were consistently above 0.6 (Fig. 5H). In conclusion, the nomogram integrating clinical features and risk scores offers a valuable tool for personalized survival prediction in patients with LUAD.

### 3.5. Correlation between immune microenvironment and prognostic genes

**Figure 6.**
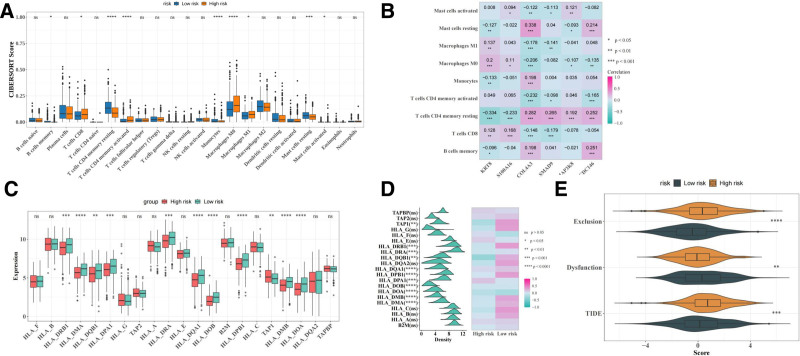
Immune infiltration and immune escape analysis. (A) Differences in 22 immune cell types between risk groups. ns; *P < .05; ***P < .001; and ****P < .0001. (B) Heatmap showing the connection between differential immune cells and prognostic genes. ns; *P < .05; **P < .01; and ***P < .001. (C) Differences of 21 MHC gene expressions between risk groups. ns; **P < .01; ***P < .001; and ****P < .0001. (D) Heatmap illustrating MHC gene expressions across risk groups. ns; *P < .05; **P < .01;***P < .001; and ****P < .0001. (E) Differences in TIDE, Dysfunction, Exclusion scores between risk groups. **P < .01; ***P < .001; and ****P < .0001. MHC = major histocompatibility complex, ns = not significant.

Immune cell infiltration, MHC gene variation, and immune escape potential were analyzed to assess differences in the immune microenvironment between the low- and high-risk groups. The results of immune infiltration between the high-risk and low-risk groups showed that 9 out of 22 immune cells had significant differences (*P*-value < .05). These cells were B cells memory, T cells CD8, T cells CD4 memory resting, T cells CD4 memory activated, monocytes, macrophages M0, macrophages M1, mast cells resting, and mast cells activated (Fig. 6A). The results of the correlation analysis showed that there were significant positive and negative correlations between different types of immune cells and genes. Among them, mast cells resting and COL4A3 exhibited the strongest positive correlation, while T cells CD4 memory resting and KRT8 showed the most significant negative correlation. In addition, other cells also had significant correlations (Fig. 6B). Furthermore, the variability of the 21 MHC genes was analyzed (Fig. 6C, D). TIDE scoring was employed to further evaluate immune escape potential across different risk groups, with the results indicating higher TIDE scores in the high-risk group compared to the low-risk group (Fig. 6E). Notably, exclusion and eysfunction scores also exhibited significant differences. These results suggest that alterations in the immune microenvironment may substantially impact the prognosis of patients with LUAD.

### 3.6. TMB analysis and chemotherapy drug sensitivity evaluation

**Figure 7.**
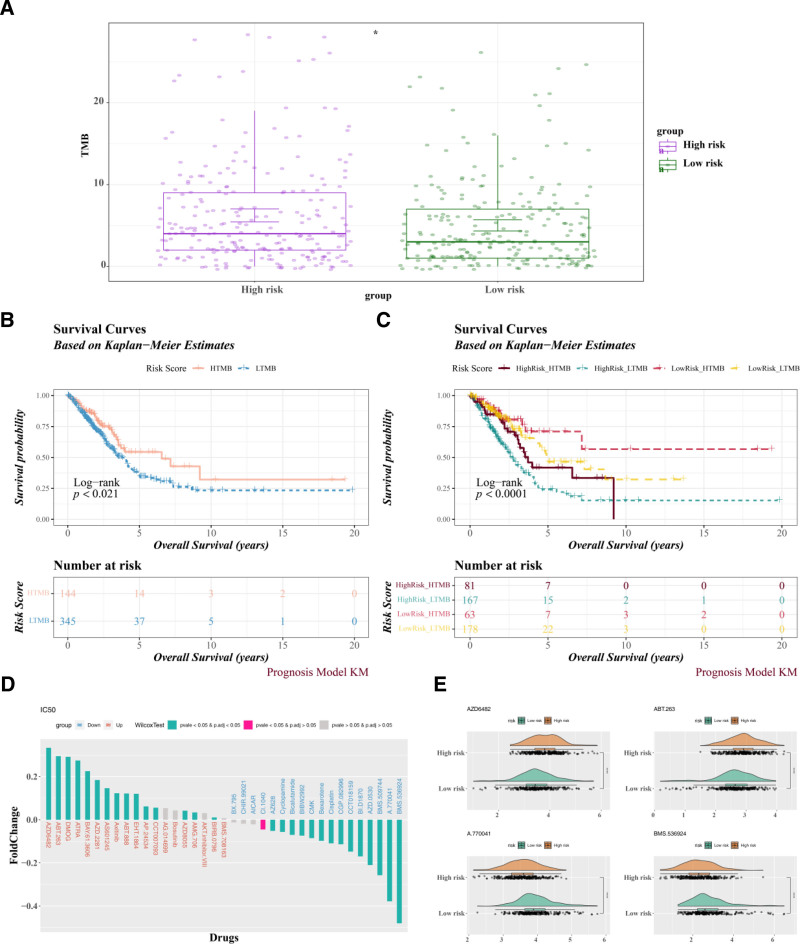
TMB and drug sensitivity analyses. (A) TMB scores differences between risk groups. Each scatter point represents the TMB value of an independent sample, and the horizontal line and error bar indicate the median TMB and standard error (median ± SE) of each group, respectively. *P < .05. (B, C) K–M survival curves for low and high TMB groups (B), as well as for combined risk-TMB groups (C). (D) Differences in drug IC_50_ values between risk groups. (E) Top 2 drugs with the most significant positive and negative IC_50_ differences. IC_50_ = half-maximal inhibitory concentration, K–M = Kaplan–Meier, SE = standard error, TMB = tumor mutational burden.

TMB scores for all patients in the TCGA-LUAD dataset were calculated to investigate the association between TMB and risk scores. The results showed significantly higher TMB scores in the high-risk group than in the low-risk group (Fig. 7A). K–M survival analysis demonstrated a significant correlation between TMB scores and patient prognosis (Fig. 7B). Patients were subsequently stratified into 4 subgroups based on TMB and risk scores: HighRisk-HTMB, HighRisk-LTMB, LowRisk-HTMB, and LowRisk-LTMB. Among these, the HighRisk-LTMB subgroup exhibited the lowest survival rate (Fig. 7C). Based on the chemotherapy drug sensitivity of the high-risk group, a strong correlation was found between the risk score and 30 chemotherapy drugs (Fig. 7D). Four potential chemotherapeutic agents for LUAD were identified: AZD6482, ABT-263, A-770041, and BMS-536924 (Fig. 7E). In summary, TMB scores were closely linked to prognosis, and combining them with risk scores improved prognostic accuracy. The identified chemotherapy drugs offer promising targets for personalized LUAD treatment.

### 3.7. Gene mutation analysis

**Figure 8.**
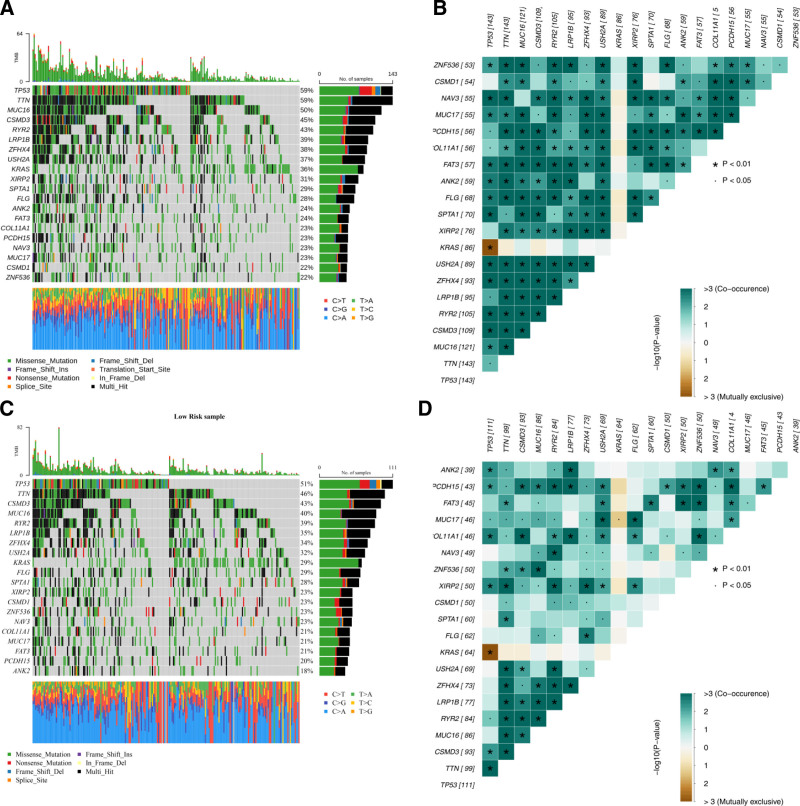
Mutation analysis in risk groups. (A, B) Top 20 mutated genes in the high-risk group (A) and their correlation analysis (B). (C, D) Top 20 mutated genes in the low-risk group (C) and their correlation analysis (D). A point *P* < .05; *P < .01.

The mutation status of the top 20 most frequently mutated genes was evaluated in the low- and high-risk LUAD groups. In the high-risk group, TTN and TP53 were the most frequently mutated genes, each with a mutation rate of 59% (Fig. 8A). The results of the correlation analysis in the high-risk group showed that there was a significant negative correlation between the mutant genes TP53 and KRAS, and the top 20 mutant genes showed an overall positive correlation (Fig. 8B). In the low-risk group, TTN and TP53 had mutation rates of 51% and 46%, respectively, among the top 20 mutated genes (Fig. 8C).In the low-risk group, TP53 and KRAS also show a significant negative correlation, and there is a certain correlation among the top 20 mutant genes (Fig. 8D). These findings provide valuable insights for further research into the biological mechanisms underlying gene mutations in LUAD.

### 3.8. GSEA enrichment analysis of prognostic genes

**Figure 9.**
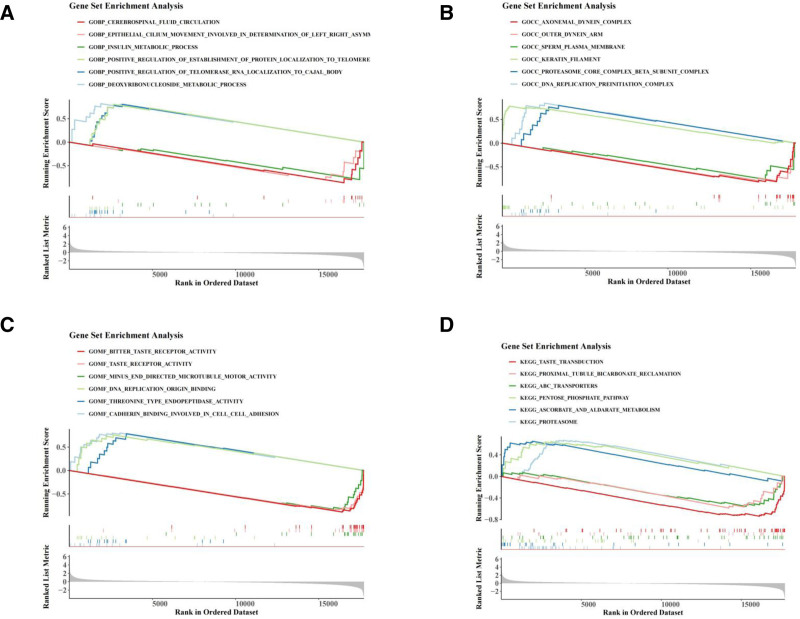
GSEA enrichment analysis in different risk groups based on GO and KEGG databases. (A–D) BP, (A), CC (B), and MF (C) pathways, as well as KEGG pathways (D) enriched in differential genes between risk groups. BP = biological process, CC = cellular component, GO = gene ontology, GSEA = gene set enrichment analysis, KEGG = Kyoto Encyclopedia of Genes and Genomes, MF = molecular function.

GSEA, utilizing GO and KEGG background gene sets, was performed to investigate the functional roles of prognostic genes and their involvement in signaling pathways. In BPs, the prognostic genes were primarily associated with the positive regulation of telomerase RNA localization to the Cajal body and the deoxyribonucleoside metabolic process (Fig. 9A). In terms of CCs, these genes were enriched in activities related to cadherin binding, extracellular matrix structure, and cell adhesion (Fig. 9B). Their main molecular functions included cadherin binding, threonine-type endopeptidase activity, and cell–cell adhesion (Fig. 9C). KEGG pathway analysis (Fig. 9D) highlighted the involvement of these genes in pathways such as proteasome regulation, ascorbate, and aldonate metabolism. These results suggest that prognostic genes are pivotal regulators of cell adhesion and metabolism, potentially influencing LUAD initiation, progression, and prognosis.

### 3.9. Expression analysis and validation of key genes

**Figure 10.**
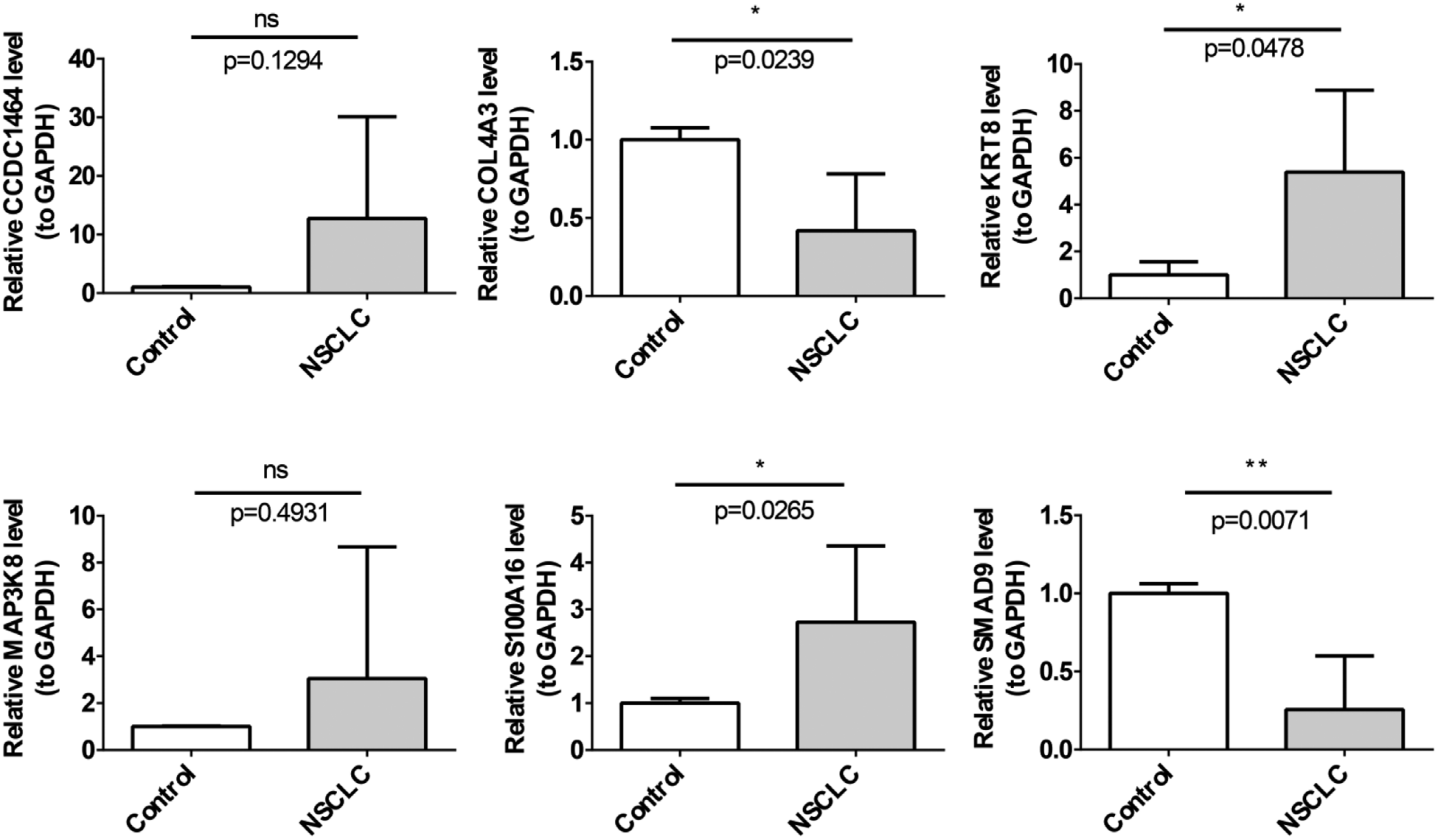
The qRT-PCR validation of KRT8, S100A16, COL4A3, SMAD9, MAP3K8, and CCDC146 expression in tumor and adjacent normal tissues. ns; *P < .05; the error line represents standard deviation (SD). ns = not significant, qRT-PCR = quantitative real time polymerase chain reaction, SD = standard deviation.

To further validate these findings, qRT-PCR analysis was performed, revealing that KRT8 and S100A16 were considerably upregulated in tumor samples, whereas COL4A3 and SMAD9 were significantly underexpressed. However, the expression levels of MAP3K8 and CCDC146 did not show significant differences (Fig. 10).

## 4. Discussion

LUAD, the most common subtype of NSCLC, is associated with a low survival rate.^[38]^ The challenge in treating LUAD is exacerbated by immune escape mechanisms and tumor heterogeneity. Therefore, identifying key genes involved in immune escape and CAF activity is essential for enhancing LUAD diagnosis, prognosis, and treatment strategies. This study utilized the TCGA-LUAD and GSE72094 datasets to identify 6 core genes (*KRT8, S100A16, COL4A3, SMAD9, MAP3K8, and CCDC146*) associated with immune escape and CAF activity in LUAD, and developed a robust risk prediction model. The model demonstrated strong predictive performance in survival and drug sensitivity analyses, and its robustness was further validated using an external validation set. These findings provide valuable insights into the molecular mechanisms underlying LUAD and establish a foundation for identifying new therapeutic targets.

Immune escape is a critical mechanism by which tumor cells evade immune detection and attack. Well-established immune escape mechanisms include CTLA-4, TIGIT, and TIM-3, which mediate immune response inhibition through immune checkpoint receptors. Additional factors such as VISTA, expressed on T and NK cells, and LAG-3, contribute to immune evasion. In the TME of patients with LUAD, immune escape often involves the overexpression of immune checkpoint molecules and the accumulation of immunosuppressive cells.^[39]^ Single-cell RNA sequencing of EGFR-mutant LUAD tumors has shown high PD-L1 expression, which is closely linked to the accumulation of M2 macrophages and regulatory T cells.^[40]^ CAFs further promote immune suppression and PD-L1 upregulation by secreting IL-6 and TGF-β, accelerating immune escape.^[40]^ Liu et al indicated that IL-6 and CCL2 may regulate T cell proliferation by modulating their activity. When CCL2 binds to its receptors on T cells, it can increase the expression of PD-1/PD-L1, thereby influencing treatment efficacy.^[41]^ Zhu’s study demonstrated that patients with LUAD exhibiting high neutrophil content had significantly worse prognosis, potentially due to the more active BPs linked to tumor progression.^[42]^ Liu XL developed an NRG-based prognostic model for LUAD, exploring its influence on tumorigenesis and progression through the SNHG14/hsa-miR-101-3p/KL/PLK1 signaling axis. This model could serve as a valuable prognostic tool to predict clinical outcomes and inform personalized therapeutic strategies.^[43]^

This study identified 183 DE-IE-CAF-RGs associated with immune escape and CAF activity, involved in key BPs and signaling pathways, including immune regulation, transmembrane transport, and glucose metabolism. Further analysis pinpointed 6 critical prognostic genes: *KRT8, S100A16, COL4A3, SMAD9, MAP3K8, and CCDC146. KRT8*, located on chromosome 12, is a member of the type II keratin family, encoding intermediate filament proteins essential for maintaining cellular structural integrity.^[44]^ Highly expressed in LUAD, KRT8 is associated with poor prognosis. Knockdown of KRT8 significantly inhibited LUAD cell migration and invasion.^[45]^ S100A16, part of the S100 protein family, activates the PI3K/AKT pathway and promotes tumor cell migration and invasion.^[46]^ In patients with NSCLC, elevated S100A16 expression is also linked with poor prognosis.^[47]^ Jia et al utilized clinical data from LUAD patients in the TCGA database to screen IERGs, identifying CEP55 and AHSA1 as prognostic and therapeutic targets for LUAD.^[48]^ The research team analyzed the tumor immune status of LUAD patients by estimating the proportions of stromal and immune cells in malignant tissues. They also measured protein expression levels of DEGs in lung cancer samples using the Human Protein Atlas database.^[48]^ Our study revealed DE-IE-CAF-RGs in LUAD. We collected samples from newly diagnosed LUAD patients and performed RNA expression analysis on LUAD tumors and adjacent normal tissues using qRT-PCR. Furthermore, we validated the risk model through in vivo experiments. In our analysis, both KRT8 and S100A16 were upregulated in LUAD tumor tissues, reinforcing their roles in LUAD progression and highlighting their potential as immune-related therapeutic targets.

Using the 6 identified genes, a risk prediction model was developed and validated through prognostic stratification, drug sensitivity analysis, and evaluation of immunotherapy potential. The model successfully distinguished high-risk and low-risk patients, with K–M survival analysis showing significantly reduced survival rates in the high-risk group. Drug sensitivity analysis identified several potential drugs for patients with LUAD, including AZD6482, ABT-263, A-770041, and BMS-536924. Immune infiltration analysis revealed a notable reduction in CD8+ and memory T cells in high-risk patients, coupled with an increased TIDE score. Tumors in high-risk patients exhibited a weaker immune response, potentially compromising the effectiveness of immunotherapy. Moreover, high-risk patients with low TMB showed significantly worse survival outcomes, whereas elevated TMB was generally associated with better responses to immunotherapy and a higher neoantigen load.^[49]^ Integrating TMB scores with the risk model presents new strategies and insights for personalized immunotherapy in LUAD.

While this study provides valuable insights into prognostic evaluation and immunotherapy strategies for LUAD, some limitations must be acknowledged. Compared to the IERGs (such as CEP55 and AHSA1) screened by Jia et al,^[48]^ this study integrated IERGs and CAFRGs, identifying key genes like KRT8 and S100A16 that simultaneously regulate both mechanisms through WGCNA. This multi-omics cross-validation approach enhances the reliability of the model and demonstrates greater synergy compared to single-mechanism analyses.^[41,43,48]^ The established gene risk model was validated in both TCGA and gene expression omnibus cohorts (AUC > 0.6) and was further associated with TIDE scores and drug sensitivity (e.g., AZD6482), providing a basis for chemotherapy selection in patients resistant to immunotherapy. Additionally, the model relies on qRT-PCR-detectable genes, making it more feasible for resource-limited settings compared to whole-genome sequencing. CIBERSORT analysis revealed that high-risk cases exhibited reduced CD8+ T cells and enhanced immune escape, complementing Zhu et al ‘s findings on neutrophils and collectively advancing the understanding of the LUAD immune microenvironment.^[42]^ However, certain limitations remain. First, the small sample size in the qRT-PCR validation may have impacted the accuracy of the results. To enhance robustness, the risk model should be further validated in larger, multicenter cohorts. Second, future studies should investigate the regulatory mechanisms and specific functions of the identified key genes, particularly their roles in immune escape and metabolic reprogramming. Lastly, further research is needed to explore how the risk model can be integrated into clinical decision-making to guide personalized treatment strategies, ultimately improving LUAD prognosis and optimizing immunotherapy efficacy.

## 5. Conclusion

This study explored LUAD prognostic genes related to immune evasion and CAFs by integrating data from public databases and applying multiple bioinformatics approaches. A novel 6-gene risk model comprising *KRT8, S100A16, COL4A3, SMAD9, MAP3K8, and CCDC146* was developed, effectively stratifying patients into high-risk and low-risk groups. CIBERSORT and TIDE analyses showed that high-risk patients had reduced infiltration of CD8+ T cells and memory B cells, elevated immune escape potential, and shorter survival, highlighting the strong relationship between the immune microenvironment and prognosis. Additionally, drug sensitivity analysis identified potential therapeutic drugs. This study provides a new prognostic model for assessing LUAD prognosis and guiding immunotherapy, offering significant clinical application potential.

## Acknowledgments

We thank Dr XueNa Wang, Dr Rui Geng, and Dr Yuan Hao for technical support. We thank Dr Xu Li and Dr Bin Song for their helpful suggestions and discussion.

## Author contributions

**Conceptualization:** Wen Su.

**Investigation:** Rui Geng, Yuan Hao.

**Methodology:** Xu Li, Yuan Hao.

**Project administration:** Rui Geng, Wen Su.

**Resources:** Xu Li, Rui Geng, Yuan Hao.

**Software:** Xu Li, XueNa Wang.

**Supervision:** Bin Song.

**Visualization:** Bin Song.

**Writing – original draft:** Yuhui Ma.

**Writing – review & editing:** Yuhui Ma.

## Supplementary Material



## References

[1] Bray F Laversanne M Sung H. Global cancer statistics 2022: GLOBOCAN estimates of incidence and mortality worldwide for 36 cancers in 185 countries. CA Cancer J Clin. 2024;74:229–63. 38572751 10.3322/caac.21834

[2] Siegel RL Giaquinto AN Jemal A. Cancer statistics, 2024. CA Cancer J Clin. 2024;74:12–49. 38230766 10.3322/caac.21820

[3] Coudray N Ocampo PS Sakellaropoulos T. Classification and mutation prediction from non-small cell lung cancer histopathology images using deep learning. Nat Med. 2018;24:1559–67. 30224757 10.1038/s41591-018-0177-5 PMC9847512

[4] Sato M Shames DS Gazdar AF Minna JD. A translational view of the molecular pathogenesis of lung cancer. J Thorac Oncol. 2007;2:327–43. 17409807 10.1097/01.JTO.0000263718.69320.4c

[5] Doll KM Rademaker A Sosa JA. Practical guide to surgical data sets: Surveillance, Epidemiology, and End Results (SEER) database. JAMA Surg. 2018;153:588–9. 29617544 10.1001/jamasurg.2018.0501

[6] Kovács SA Győrffy B. Transcriptomic datasets of cancer patients treated with immune-checkpoint inhibitors: a systematic review. J Transl Med. 2022;20:249. 35641998 10.1186/s12967-022-03409-4 PMC9153191

[7] Wei X Li X Hu S Cheng J Cai R. Regulation of ferroptosis in lung adenocarcinoma. Int J Mol Sci. 2023;24:14614. 37834062 10.3390/ijms241914614 PMC10572737

[8] Jhunjhunwala S Hammer C Delamarre L. Antigen presentation in cancer: insights into tumour immunogenicity and immune evasion. Nat Rev Cancer. 2021;21:298–312. 33750922 10.1038/s41568-021-00339-z

[9] Hayashi K Nikolos F Lee YC. Tipping the immunostimulatory and inhibitory DAMP balance to harness immunogenic cell death. Nat Commun. 2020;11:6299. 33288764 10.1038/s41467-020-19970-9 PMC7721802

[10] Cui Y Li J Zhang P. B4GALT1 promotes immune escape by regulating the expression of PD-L1 at multiple levels in lung adenocarcinoma. J Exp Clin Cancer Res. 2023;42:146. 37303063 10.1186/s13046-023-02711-3 PMC10259029

[11] Zhu J Fan Y Xiong Y. Delineating the dynamic evolution from preneoplasia to invasive lung adenocarcinoma by integrating single-cell RNA sequencing and spatial transcriptomics. Exp Mol Med. 2022;54:2060–76. 36434043 10.1038/s12276-022-00896-9 PMC9722784

[12] Ma T Renz BW Ilmer M. Myeloid-derived suppressor cells in solid tumors. Cells. 2022;11:310. 35053426 10.3390/cells11020310 PMC8774531

[13] Biffi G Tuveson DA. Diversity and biology of cancer-associated fibroblasts. Physiol Rev. 2021;101:147–76. 32466724 10.1152/physrev.00048.2019 PMC7864232

[14] Liu T Han C Fang P. Cancer-associated fibroblast-specific lncRNA LINC01614 enhances glutamine uptake in lung adenocarcinoma. J Hematol Oncol. 2022;15:141. 36209111 10.1186/s13045-022-01359-4 PMC9548164

[15] Li Y Yang Y Ma Q. HNRNPK/CLCN3 axis facilitates the progression of LUAD through CAF-tumor interaction. Int J Biol Sci. 2022;18:6084–101. 36439880 10.7150/ijbs.76083 PMC9682536

[16] Geng X Chen H Zhao L. Cancer-Associated Fibroblast (CAF) heterogeneity and targeting therapy of CAFs in pancreatic cancer. Front Cell Dev Biol. 2021;9:655152. 34336821 10.3389/fcell.2021.655152 PMC8319605

[17] Pramesh CS Badwe RA Bhoo-Pathy N. Priorities for cancer research in low- and middle-income countries: a global perspective. Nat Med. 2022;28:649–57. 35440716 10.1038/s41591-022-01738-x PMC9108683

[18] Lu H Zheng LY Wu LY Chen J Xu N Mi SC. The immune escape signature predicts the prognosis and immunotherapy sensitivity for pancreatic ductal adenocarcinoma. Front Oncol. 2022;12:978921. 36147906 10.3389/fonc.2022.978921 PMC9486201

[19] Tan P Li M Liu Z Li T Zhao L Fu W. Glycolysis-related LINC02432/Hsa-miR-98-5p/HK2 axis inhibits ferroptosis and predicts immune infiltration, tumor mutation burden, and drug sensitivity in pancreatic adenocarcinoma. Front Pharmacol. 2022;13:937413. 35795552 10.3389/fphar.2022.937413 PMC9251347

[20] D’Arrigo G Leonardis D ElHafeez SA Fusaro M Tripepi G Roumeliotis S. Methods to analyse time-to-event data: the kaplan-meier survival curve. Oxid Med Cell Longevity. 2021;2021:2290120. 10.1155/2021/2290120 PMC8478547 34594473

[21] Lin W Wang Y Chen Y Wang Q Gu Z Zhu Y. Role of calcium signaling pathway-related gene regulatory networks in ischemic stroke based on multiple WGCNA and single-cell analysis. Oxid Med Cell Longevity. 2021;2021:8060477. 10.1155/2021/8060477 PMC8720592 34987704

[22] Yan C Niu Y Li F Zhao W Ma L. System analysis based on the pyroptosis-related genes identifies GSDMC as a novel therapy target for pancreatic adenocarcinoma. J Transl Med. 2022;20:455. 36199146 10.1186/s12967-022-03632-z PMC9533512

[23] Yu G Wang LG Han Y He QY. clusterProfiler: an R package for comparing biological themes among gene clusters. OMICS J Integr Biol. 2012;16:284–7. 10.1089/omi.2011.0118 PMC3339379 22455463

[24] Martino E Chiarugi S Margheriti F Garau G. Mapping, structure and modulation of PPI. Front Chem. 2021;9:718405. 34692637 10.3389/fchem.2021.718405 PMC8529325

[25] Friedman J Hastie T Tibshirani R. Regularization paths for generalized linear models via coordinate descent. J Stat Softw. 2010;33:1–22. 20808728 PMC2929880

[26] Subramanian A Tamayo P Mootha VK. Gene set enrichment analysis: a knowledge-based approach for interpreting genome-wide expression profiles. Proc Natl Acad Sci USA. 2005;102:15545–50. 16199517 10.1073/pnas.0506580102 PMC1239896

[27] Gu Z Hübschmann D. Make interactive complex heatmaps in R. Bioinformatics (Oxford, England). 2022;38:1460–2. 34864868 10.1093/bioinformatics/btab806 PMC8826183

[28] Wu Z Guan Q Han X. A novel prognostic signature based on immune-related genes of diffuse large B-cell lymphoma. Aging (Milano). 2021;13:22947–62. 10.18632/aging.203587 PMC8544299 34610582

[29] Iasonos A Schrag D Raj GV Panageas KS. How to build and interpret a nomogram for cancer prognosis. J Clin Oncol. 2008;26:1364–70. 18323559 10.1200/JCO.2007.12.9791

[30] Yang X Lei P Huang L Tang X Wei B Wei H. Prognostic value of LRRC4C in colon and gastric cancers correlates with tumour microenvironment immunity. Int J Biol Sci. 2021;17:1413–27. 33867855 10.7150/ijbs.58876 PMC8040466

[31] Arghittu A Deiana G Dettori M. Web-based analysis on the role of digital media in health communication: the experience of VaccinarSinSardegna Website. Acta Bio Medica. 2021;92:e2021456. 10.23750/abm.v92iS6.12072 PMC8851010 34739476

[32] Yan T Zhu S Shi Y. Pan-cancer analysis of atrial-fibrillation-related innate immunity gene ANXA4. Front Cardiovasc Med. 2021;8:713983. 34540918 10.3389/fcvm.2021.713983 PMC8446278

[33] Sun H Long J Zuo B. Development and validation of a selenium metabolism regulators associated prognostic model for hepatocellular carcinoma. BMC Cancer. 2023;23:451. 37202783 10.1186/s12885-023-10944-w PMC10197375

[34] Jardim DL Goodman A de Melo Gagliato D Kurzrock R. The challenges of tumor mutational burden as an immunotherapy biomarker. Cancer Cell. 2021;39:154–73. 33125859 10.1016/j.ccell.2020.10.001 PMC7878292

[35] Wang W Lu Z Wang M. The cuproptosis-related signature associated with the tumor environment and prognosis of patients with glioma. Front Immunol. 2022;13:998236. 36110851 10.3389/fimmu.2022.998236 PMC9468372

[36] Xu Q Chen S Hu Y Huang W. Landscape of immune microenvironment under immune cell infiltration pattern in breast cancer. Front Immunol. 2021;12:711433. 34512634 10.3389/fimmu.2021.711433 PMC8429934

[37] Gu X Lai D Liu S. Hub genes, diagnostic model, and predicted drugs related to iron metabolism in Alzheimer’s Disease. Front Aging Neurosci. 2022;14:949083. 35875800 10.3389/fnagi.2022.949083 PMC9300955

[38] Spella M Stathopoulos GT. Immune resistance in lung adenocarcinoma. Cancers. 2021;13:384. 33494181 10.3390/cancers13030384 PMC7864325

[39] Huang Z Chen X Wang Y. SLC7A11 inhibits ferroptosis and downregulates PD-L1 levels in lung adenocarcinoma. Front Immunol. 2024;15:1372215. 38655266 10.3389/fimmu.2024.1372215 PMC11035808

[40] Yang L He YT Dong S. Single-cell transcriptome analysis revealed a suppressive tumor immune microenvironment in EGFR mutant lung adenocarcinoma. J ImmunoTher Cancer. 2022;10:e003534. 35140113 10.1136/jitc-2021-003534 PMC8830346

[41] Liu Y Zhou J Wu J. Construction and validation of a novel immune-related gene pairs-based prognostic model in lung adenocarcinoma. Cancer Control. 2023;30:10732748221150227. 36625357 10.1177/10732748221150227 PMC9834935

[42] Zhu Q Chai Y Jin L. Construction and validation of a novel prognostic model of neutrophil‑related genes signature of lung adenocarcinoma. Sci Rep. 2023;13:18226. 37880277 10.1038/s41598-023-45289-8 PMC10600204

[43] Liu X Li X Shen X Ma R Wang Z Hu Y. Construction of a prognostic model for lung adenocarcinoma based on necroptosis genes and its exploration of the potential for tumor immunotherapy. Transl Cancer Res. 2025;14:2563–79. 40530113 10.21037/tcr-24-2165 PMC12170285

[44] Xie L Dang Y Guo J. High KRT8 expression independently predicts poor prognosis for lung adenocarcinoma patients. Genes. 2019;10:36. 30634629 10.3390/genes10010036 PMC6360019

[45] Chen H Chen X Pan B Zheng C Hong L Han W. KRT8 serves as a novel biomarker for LUAD and promotes metastasis and EMT via NF-κB signaling. Front Oncol. 2022;12:875146. 35664775 10.3389/fonc.2022.875146 PMC9160746

[46] Wu C Yang J Lin X Li R Wu J. miR-508-5p serves as an anti-oncogene by targeting S100A16 to regulate AKT signaling and epithelial-mesenchymal transition process in lung adenocarcinoma cells. Am J Med Sci. 2023;365:520–31. 36967030 10.1016/j.amjms.2023.02.014

[47] Katono K Sato Y Kobayashi M. S100A16, a promising candidate as a prognostic marker for platinum-based adjuvant chemotherapy in resected lung adenocarcinoma. OncoTargets Ther. 2017;10:5273–9. 10.2147/OTT.S145072 PMC5679695 29138580

[48] Jia HR Li WC Wu L. The prognostic value of immune escape-related genes in lung adenocarcinoma. Transl Cancer Res. 2024;13:2647–61. 38988926 10.21037/tcr-23-2295 PMC11231773

[49] Ding D Wang L Zhang Y Shi K Shen Y. Machine learning developed a programmed cell death signature for predicting prognosis and immunotherapy benefits in lung adenocarcinoma. Transl Oncol. 2023;38:101784. 37722290 10.1016/j.tranon.2023.101784 PMC10511492

