# Supplementary material for: Identification and validation of a diagnostic and prognostic model based on immune escape and cancer-associated fibroblast-related genes in lung adenocarcinoma
Source: Medicine (Baltimore). 2025 Nov 14;104(46):e45756. doi: 10.1097/MD.0000000000045756 (PMC12622665; doi:10.1097/MD.0000000000045756)
Supplement: Supplementary file 1 [file medi-104-e45756-s001.doc]

**Supplementary Table 1**

| **Primer** | **Sequence** |
| --- | --- |
| KRT8 : F | AGCTTCTCCGCTCCTTCTAGG |
| KRT8 : R | CAGGCTCTGGTTGACCGTAA |
| S100A16 : F | CGGCATGGGAGTAGGAATGG |
| S100A16 : R | GGAATGTGCTCCAGTCACCC |
| COL4A3 : F | AGGAAACAGAGGCGTTCCAG |
| COL4A3 : R | AGTGCTGCCCAAATCTCCTC |
| SMAD9 : F | TTCCAAAGCACTCGCAGCTA |
| SMAD9 : R | GCATCTACAGGTTGGCCACT |
| MAP3K8 : F | GCTCTGGCTGGCTACTTCAA |
| MAP3K8 : R | CACATTCACGGGTCACAGGA |
| CCDC1464 : F | TTCCCTTAGTCCTAGTCCTGCC |
| CCDC1464 : R | GGCATCTCTGTCACCTGAGC |
| GAPDH : F | GACCCCTTCATTGACCTCAAC |
| GAPDH : R | GCCATCACGCCACAGCTTTCC |
| GAPDH : F | CCCATCACCATCTTCCAGG |
| GAPDH : R | CATCACGCCACAGTTTCCC |
